# Ultra-high resolution, multi-scale, context-aware approach for detection of small cancers on mammography

**DOI:** 10.1038/s41598-022-15259-7

**Published:** 2022-07-08

**Authors:** Krithika Rangarajan, Aman Gupta, Saptarshi Dasgupta, Uday Marri, Arun Kumar Gupta, Smriti Hari, Subhashis Banerjee, Chetan Arora

**Affiliations:** 1 School of Information Technology, Indian Institute of Technology, Delhi, India; 2 Present Address: Department of Radiology, All India Institute of Medical Sciences, New Delhi, India; 3 Department of Computer Science and Engineering, Indian Institute of Technology, Delhi, India; 4 Present Address: Department of Computer Science, Ashoka University, Sonepat, Harayana, India

**Keywords:** Oncology, Computer science, Breast cancer, Machine learning

## Abstract

While detection of malignancies on mammography has received a boost with the use of Convolutional Neural Networks (CNN), detection of cancers of very small size remains challenging. This is however clinically significant as the purpose of mammography is early detection of cancer, making it imperative to pick them up when they are still very small. Mammography has the highest spatial resolution (image sizes as high as 3328 × 4096 pixels) out of all imaging modalities, a requirement that stems from the need to detect fine features of the smallest cancers on screening. However due to computational constraints, most state of the art CNNs work on reduced resolution images. Those that work on higher resolutions, compromise on global context and work at single scale. In this work, we show that resolution, scale and image-context are all important independent factors in detection of small masses. We thereby use a fully convolutional network, with the ability to take any input size. In addition, we incorporate a systematic multi-scale, multi-resolution approach, and encode image context, which we show are critical factors to detection of small masses. We show that this approach improves the detection of cancer, particularly for small masses in comparison to the baseline model. We perform a single institution multicentre study, and show the performance of the model on a diagnostic mammography dataset, a screening mammography dataset, as well as a curated dataset of small cancers < 1 cm in size. We show that our approach improves the sensitivity from 61.53 to 87.18% at 0.3 False Positives per Image (FPI) on this small cancer dataset. Model and code are available from https://github.com/amangupt01/Small_Cancer_Detection

## Introduction

Breast cancer is the most common cancer in women, and the second most common cancer overall in the world^1^. Early detection is a crucial factor that aids in reducing mortality rates due to breast cancer^2^. Mammography is therefore offered as a screening modality for women over a certain age in many countries for early detection of cancer^3^. In fact, the 10 year survival rate due to breast cancer falls from over 95% in patients where the cancer is less than 1 cm to about 60% when the size of the cancer is over 3 cm^4^. However, detecting cancer on mammograms is a tedious job^5^, apart from requiring highly specialized breast radiologists. Only about 5 out of 1000 scans would harbor a cancer^6^. The cancer may also only occupy less than 1% of the image. The ability to identify these tiny cancers which are likely to be missed by a fatigued radiologist would therefore be an important contribution of computer vision.

**Figure 1:**
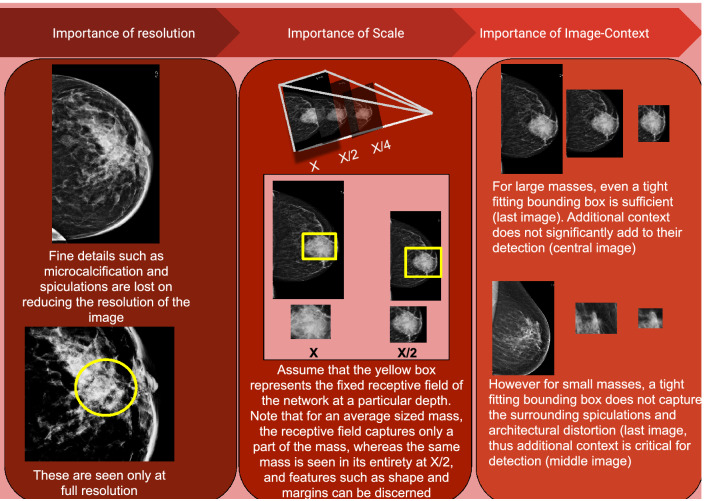
Importance of resolution, scale and Image-context in detection of small cancers on mammography.

State-of-art deep learning based image classifiers^7,8^ and object detectors^9–11^ have performed exceedingly well in mammography. The reader is directed to the systematic review by Freeman et al.^12^ for a more detailed description of currently available deep learning tools. However the sensitivity for detection of small masses is lower than for large masses^13^. Recently, some authors have attempted to design approaches specific to small cancer detection. Savelli et al.^14^ in their work designed a multi-context network for small lesion detection, however they show their results only on microcalcification. Agarwal et al.^13^ trained a patch-based classifier while showing the benefit of domain adaptation in mammography. They showed their results on masses of different sizes in their analysis, showing that the performance of CNNs drops for small masses. Lotter et al.^15^ perform multi-scale curriculum learning on mammograms to deal with the problem of very small lesions in comparison to image size. However, they perform only image level classification, without precise cancer localisation. In this work, we deliver a concerted effort towards the detection of small sized cancers, which represent a clinically significant problem. We analyze this problem from the perspective of mammography and propose a solution by combining resolution, scale as well as image-context. (Fig. 1).

### Importance of spatial resolution

Fine Microcalcifications and details such as spiculations are central to identifying breast cancers, the visibility of which on mammography is critically dependent on the spatial resolution of a mammogram. In fact, out of all imaging modalities, mammography has the highest spatial resolution. Digital Mammograms have a pixel size of around 50–100 microns^16^. DMs tend to be very large images, ranging from 2300 × 1800 pixels (of dimension 100 microns) to 4096 × 5625 pixels (of dimension 54 micron)^17^. Most CNNs however take much smaller input sizes, thus losing this information so critical to the diagnosis of small masses.

### Importance of scale

When mammograms are used in full resolution as input to a network, the receptive field typically includes only a fraction of the image. While full resolution is vital for identifying fine details (such as spiculations), masses may not be seen in its entirety within the receptive field. Thereby the shape and margins of the mass would not be visualized, which are important descriptors of any mammographic mass (Fig. 1). Thus, apart from seeing mammograms at original resolution, we propose that a multi-scale approach where the image is also seen at reduced scale would be particularly beneficial in the context of mammograms.

### Importance of image-context

The features essential for detection of small cancers, may be significantly different from those for large (or average sized) cancers, much like in case of small objects in natural images^18^. While shape, margins and density are important descriptors for average sized masses, for a very small mass, features such as margins and shape may not be visible directly. Other cues in surrounding parenchyma such as architectural distortion and the skin bulge or retraction play an important role in the detection. Thus we propose that image-context is particularly important in the case of small sized cancers.

### Approach to detection of small cancers

Though resolution, scale and context are all well described factors playing an important role in object detection, designing models which can do all these together is challenging. Our ideas and network for incorporating scale and context for detection are inspired by the work of Hu et al.^18^ on small object detection in natural images. We hereby present a concerted effort towards small cancer detection with our network, which takes into account all the above factors, without compromising on each other.

## Materials and methods

### Data

This was a single institution, multi-centre study. We show our results on one diagnostic mammography dataset, one screening mammography dataset and a curated dataset with small cancers.

#### Training dataset

For training our network, we collected a dataset from our hospital consisting of Full Field Digital Mammograms (FFDM) acquired on Selenia Dimensions, a Hologic Mammography unit from Jan 2015 to Dec 2015. In order to make a balanced dataset suitable for training, we collected consecutive patients who had been assigned a BIRADS 4 category and had a histopathological diagnosis. Thus this dataset consisted of 839 images, with 393 cancers. The images of the contralateral breast provided normal examples to the network.

#### Test datasets

##### Diagnostic mammography dataset

Our country has no formal mammography screening program. Thus our test dataset (acquired from the same centre as the training dataset) had a distribution of diagnostic mammography practice. For this dataset, consecutive patients who underwent mammography from January 2018 to June 2018 were chosen. Patients who had been given a BIRADS 4 or above but did not have histopathological report were excluded from the study. There were 2569 images with 243 cancers in this dataset. This is referred to as the DM dataset in further discussion.

##### Screening mammography dataset

This was an external dataset obtained from our cancer centre, where opportunistic screening is offered to all eligible women. All patients who underwent mammography from January to April 2021 were selected, except those without histological proof for BIRADS 4 or above lesions. This dataset provided an external test dataset, as well as helped ascertain our efficacy in a screening setting. These images were acquired on a Hologic system. There were 2146 images with 59 cancers, and this is referred to as the SM dataset in further discussion.

##### Small cancer dataset

In order to establish the value of our network in small cancers, we curated a dataset of patients with cancers less than 1 cm in size (diameter of mass was used for masses and longest dimension of the cluster was used for a cluster of microcalcification). There were 79 images in this dataset with an average cancer size of 5.8 mm diameter. This is referred to as the SC dataset. Images in this dataset came from both the above centres.

All datasets were collected after obtaining ethical clearance from the Institutional Review Board (IRB) of the All India Institute of Medical Sciences with reference number IEC-247/04.05.2018. This data was de-identified, informed consent was obtained for use of data from all patients participating in the study. All experimental protocols were approved by the IRB of the All India Institute of Medical Sciences, New Delhi and all methods were carried out in accordance with prevailing guidelines and regulations. Bounding box annotations were performed by 3 breast radiologists with 2, 8 and 15 years of experience in breast imaging. All images were of size 3328 × 4096 pixels.

### Model architecture

#### Pre-processing

All input images were of size 3328 × 4096. These were initially cropped to remove the portion of the mammogram that had no breast in it. These images were thus of variable size (depending on the size of the breast) on one dimension and 4096 pixels on the other dimension. These were then passed forward in the network.

#### Network architecture

**Figure 2:**
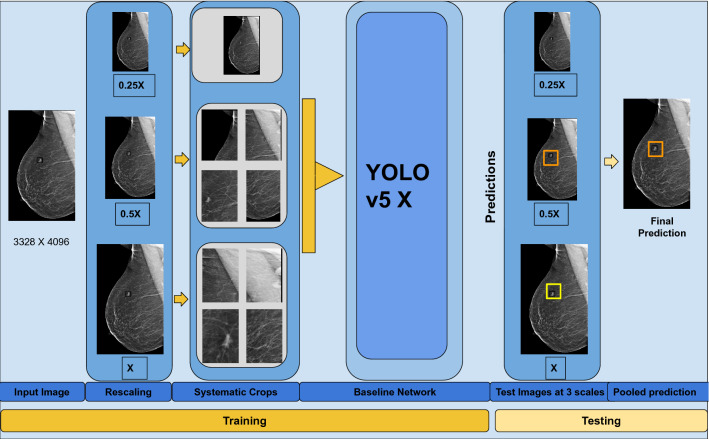
Proposed Architecture for detection of small cancers. The image is rescaled to 3 different scales, and fixed sized crops are systematically obtained to provide inputs with variable resolution, scale and context to the network. Predictions from all 3 scales are combined at test time.

Our proposed architecture is shown in Fig. 2. The network involved the following steps (1) Generating multiple scales (2) Systematic crop of images (3) Passing through baseline architecture (4) Combination at test time.

##### Generation of multiple scales

The full resolution image is rescaled to give images at 3 scales- X, 0.5X and 0.25 X, where X is the original image.

##### Systematic crops

Crops of size 0.25 times the original image are taken from all 3 scales. These crops were 1024 pixels on one dimension, and variable size on the other dimension. These crops constitute the input to the network. The crops are systematically taken from the larger images from right to left and top to bottom ensuring that no part of the image is left out.

##### Baseline architecture

We chose YOLO v5 (You Only Look Once version 5)^19^ as our baseline architecture as it is fully convolutional and thus allows us to pass images of all input sizes. Our ablation study to choose the baseline network is presented in the results section. We used YOLO v5 with a CSPDarknet backbone, a PANet neck with upsampling and Concatenations from different layers and a final YOLO head Convolution. The CSPDarknet is used for feature extraction, PANet for feature fusion and the YOLO layer for computing class and objectness scores. Concatenation of feature vectors at varying layers within the back-bone architecture helps to leverage the varying contextual information available in different layers. Each layer therefore corresponds to increasingly larger receptive fields, which is instrumental in detecting masses of very small sizes.

##### Combination of output at test time

At test time, full images were given as input in 3 scales- X, 0.5 X and 0.25X. Predictions were generated for each scale separately. These predictions were finally combined using Weighted Box Fusion^20^(WBF) as described by Wang et al. WBF uses the confidence scores from each of the models and then combines them to construct an "average” bounding box that captures the underlying ground truth boxes better than any of the individual predictions from the models. We experimented with simple Non-Maximal Suppression (NMS) and NMS with thresholds and found WBF to perform the best.

##### Implementation details

As in yolov5 default implementation, we used a Binary Cross-Entropy with Logits Loss for computation of object scores, and SGD as the Optimizer. We kept the batch size to 16, and the initial learning rate to be 0.01. All computations were carried out on High Performance Computing Cluster having 32 GB V100 GPUs.

## Results and experiments

We evaluated the model using Free-Response Operator Characteristic (FROC) curves by plotting the sensitivity of the network against the false positive marks per image. A detection was considered a true detection if the center of the predicted box fell anywhere within the ground-truth box, as is the standard practice in mammography^21,22^.

### Selection of baseline architecture

For selection of baseline network, 4 object detection networks which were fully convolutional were trained on our training data and tested on the DM dataset. The results are given in Table 1.

**Table 1:** Selection of a fully convolutional network as baseline for our proposed network.

Network	Sensitivity at 0.1 FPI	0.2 FPI	0.3 FPI
YOLO v3	0.679	0.757	0.786
YOLO v5 small	0.634	0.733	0.786
YOLO v5 large	0.638	0.708	0.770
YOLO v5 X	0.654	0.758	0.811

### Results on the DM, SM and small cancer dataset

**Figure 3:**
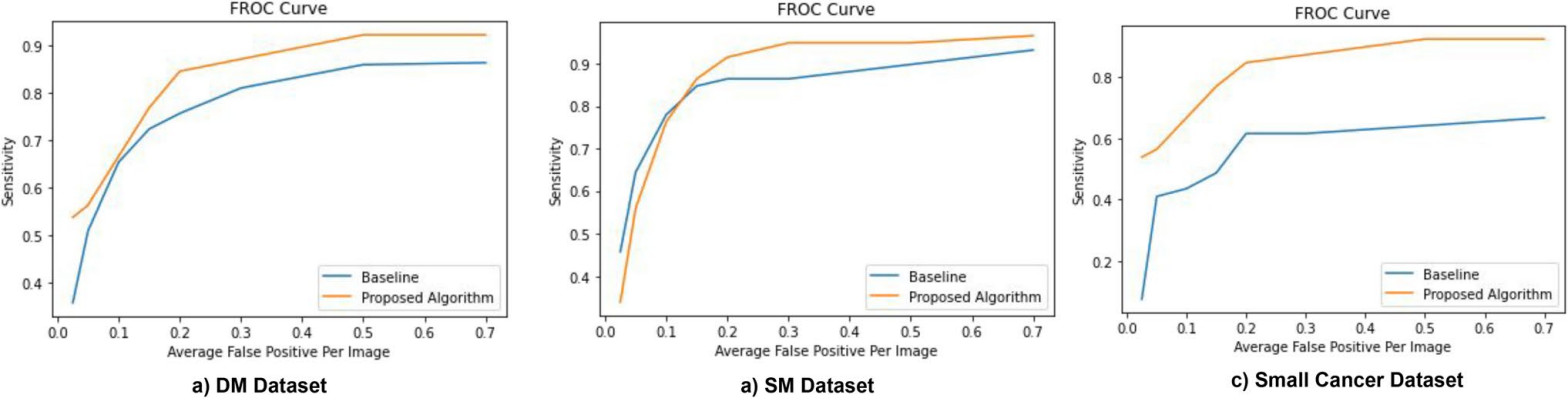
Performance of the proposed network on the DM dataset (**a**), SM dataset (**b**) and the SC dataset (**c**). Results of baseline architecture are plotted for comparison.

Our FROC curves on the DM, SM and SC datasets are given in Fig. 3a–c respectively . The results of the baseline architecture is also plotted for comparison. Table 2 summarizes the performance of the network on the 3 datasets.

**Table 2:** Summary of performance of our proposed network on the 3 datasets.

Dataset	Sensitivity at 0.15 FPI (proposed/baseline)	0.2 FPI (proposed/baseline)	0.3 FPI (proposed/baseline)
Diagnostic mammography	0.7037/*0.6543*	0.7818/*0.6831*	0.8353/*0.7201*
Screening Mammography (External Dataset)	0.8644/0.8474	0.9152/0.8644	0.9491/0.8644
Small Mass dataset	0.7692/ 0.4870	0.8461/0.6153	0.8717/0.6153

### Ablation studies

The architecture we have described has been built on 3 basic principles: resolution, scale and context. In order to study the effect of each of these components, we performed a few ablation experiments on our Small Cancer dataset (Fig. 5).

**Figure 4:**
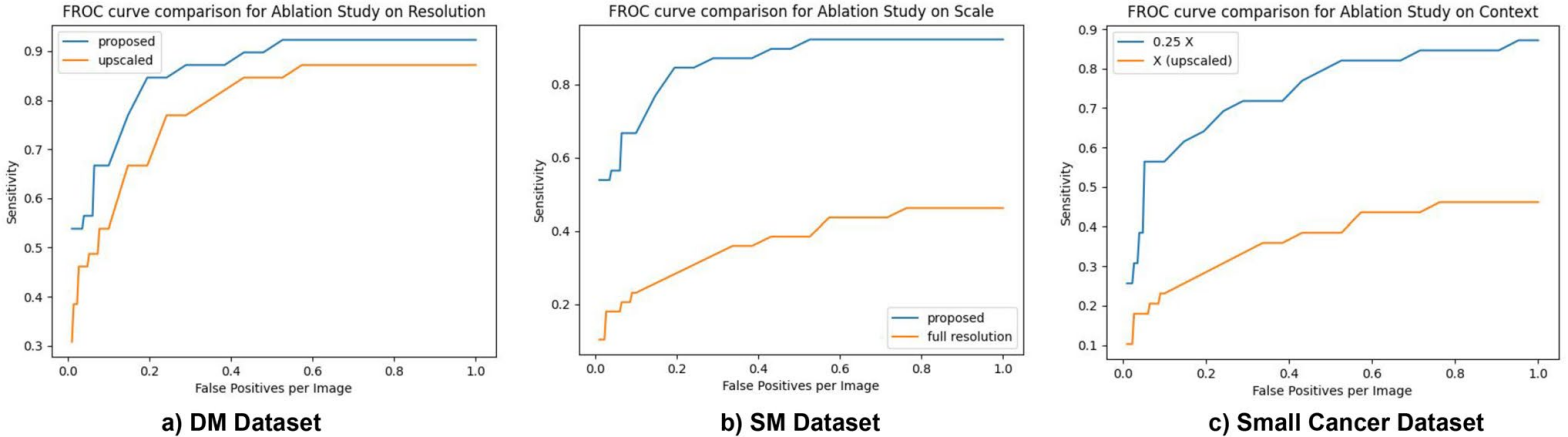
Effect of resolution (**a**), scale (**b**) and context (**c**) on performance of network.

First, to study the effect of resolution, we tested the small cancer dataset by upsampling the low resolution images instead of using higher resolutions for crops. Here the images were first downsampled to 0.25X, and then 0.5X and X were generated by upsampling the 0.25X image. Crops were then taken from the upsampled images. Figure 4a shows the FROC thus obtained and compares it with our proposed model. As seen here, the model performs much better when crops are taken from high resolution images, rather than simply from different scales, demonstrating the importance of resolution.

Second, in order to study the effect of scale, we train our baseline architecture with only full resolution images without re-scaling them (Fig. 4b). As seen in Fig. 4b, our proposed model performs better than the model trained and tested on only the high resolution image indicating the importance of a multi-scale approach.

Finally, in order to study the effect of context, we first negate the effect of resolution by using upsampled images (upsampled from 0.25X images) rather than original resolution images as input. Crops were now systematically taken from each scale. Thus here crops from 0.25X have maximum context and crops from X have least context, though all have the same resolution. The performance on 0.25X and X are thus studied in Fig. 4c. As seen here, 0.25 X performs much better than X, demonstrating the importance of image context in cancer detection.

In order to analyze the importance of each scale/ resolution in relation to size of image, we analysed the results of each individual scale prior to WBF on the SM dataset. We analysed masses exclusively caught only on that particular scale. These are summarized in Table 3.

**Table 3:** Analysis of detection performance on individual scales prior to WBF.

Scale	Sensitivity at 0.3 FPI	Average size of mass seen only at this scale (BB size) (cms)
X	0.6923	1.2
0.5X	0.8717	1.8
0.25X	0.7179	1.7

Thus we see that each factor, resolution, scale as well as context had significant independent contribution towards achieving the best accuracy using our model.

## Discussion

Context, scale and resolution have been known to play a role in small object detection in natural images. In this work we explore the role of each of these factors towards small cancer detection specific to mammography, which presents a unique problem due to the presence of very small objects (cancers) in very large images. We showed through experiments how each of these factors have an important role to play in detection of small cancers.

**Figure 5:**
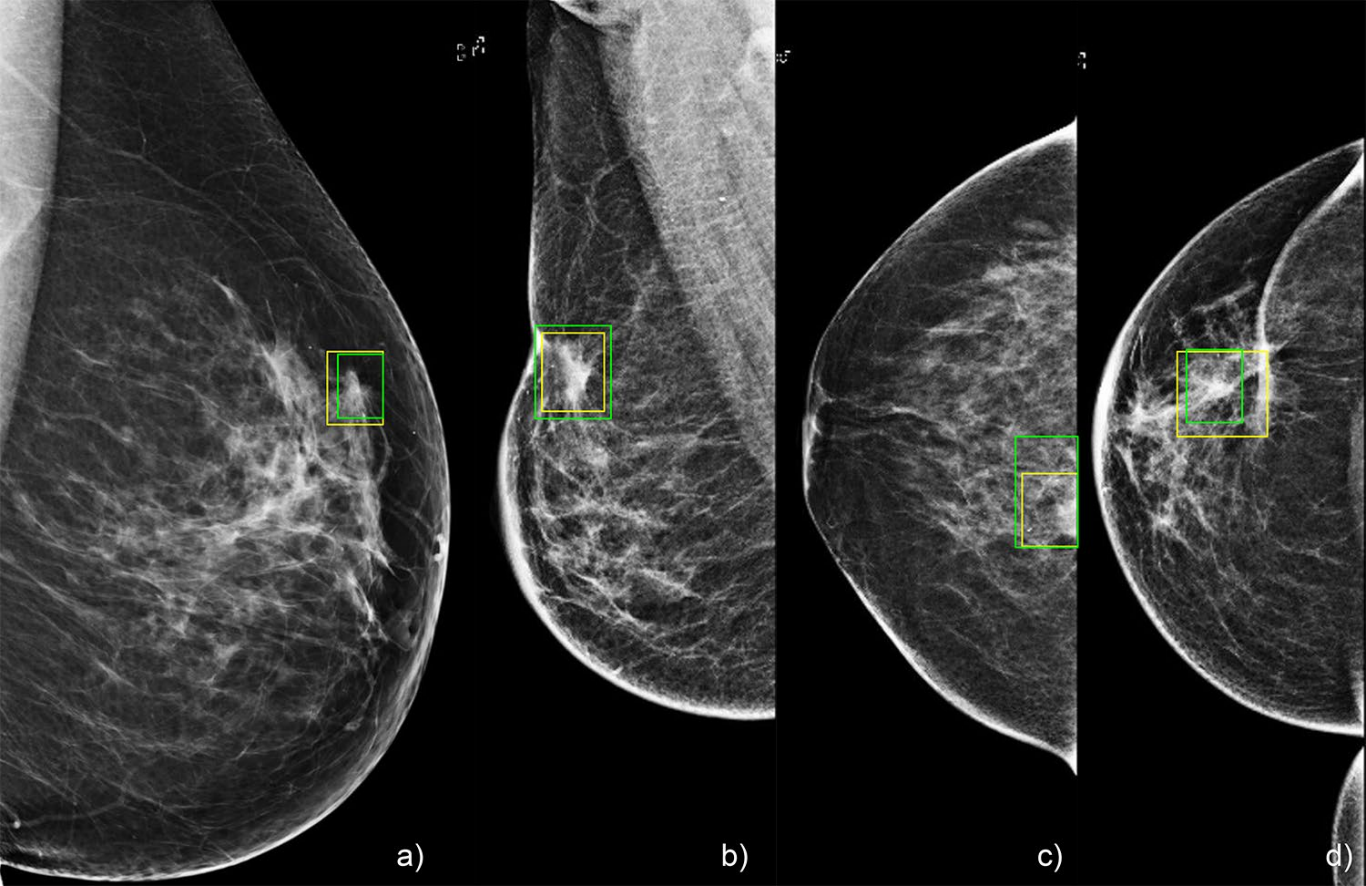
Visualization of our results of our network on small masses < 1 cm. Yellow represents the ground truth bounding box, green represents predictions of our network.

We performed a single institution, multi-centre study to validate the results of our proposed network. We validated our results on a dataset with a distribution of diagnostic mammography practice as well as a screening mammography dataset (external validation). We show that our network performs comparably in both settings. In both datasets we showed an improvement in comparison to our baseline architecture. In addition, we tested our results on a curated dataset with cancers < 1 cm in size. We show that the improvement is most marked in this dataset with our approach, suggesting that this approach is particularly suited for detection of small cancers. Some examples from this dataset which were captured by our network are given in Fig. 5. We also note that despite being an external dataset, we perform better in our SM dataset than our DM dataset. This is due to a large number of benign masses in our DM dataset which were detected as cancers, contributing to false positives in this dataset.

We analyze the importance of resolution, context and scale by comparing our performance of each factor in isolation with the performance of our proposed network. Through our ablation studies (Fig. 4) we demonstrate that each of these factors have an important role to play. Our analysis of performance of each individual scale (Table 3) also showed that though the sensitivity was the lowest at full resolution, this scale was particularly important for detection of small masses.

Our study has some limitations. We acknowledge that all mammograms came from a single vendor, thus the effectiveness of the network in other situations need further study. Analysis of masses that were missed by this network revealed that we missed isodense, obscure masses, and masses placed in the peripheral breast tissue. There were only 3 images with microcalcification clusters in the DM dataset and 9 images in the SM dataset. Therefore the performance of the network on microcalcifications has not been adequately studied. Analysis of the false positives revealed that some benign lesions, such as cysts and fibroadenomas were also detected as cancers by the network. This indicates the direction of further research in this area.

To conclude, in this work we present a concerted effort towards the detection of small cancers. In a multicentre study we show the effectiveness of our approach in the setting of diagnostic mammography, screening mammography, as well as in a subset of small cancers < 1 cms in size.

## Data Availability

All our datasets were from the All India Institute of Medical Sciences, New Delhi, and are not available publicly.
